# Changes in 5-HT1A Receptor Expression in the Oculomotor Nucleus in a Rat Model of Post-traumatic Stress Disorder

**DOI:** 10.1007/s12031-012-9874-6

**Published:** 2012-08-29

**Authors:** Dongjuan Liu, Bing Xiao, Fang Han, Feifei Luo, Enhua Wang, Yuxiu Shi

**Affiliations:** 1 Department of Electron Microscopy, College of Basic Medical Sciences, China Medical University, Shenyang, Liaoning 110001 China; 2 Department of Histology and Embryology, College of Basic Medical Sciences, China Medical University, Shenyang, Liaoning 110001 China; 3 Institute of Pathology and Pathophysiology, China Medical University, Shenyang, Liaoning 110001 China

**Keywords:** Post-traumatic stress disorder, Oculomotor nucleus, Single-prolonged stress, 5-HT1A receptor

## Abstract

Post-traumatic stress disorder (PTSD) is an anxiety disorder that develops after exposure to a life-threatening traumatic experience. Mental disorder appears after the traumatic stress incident and affects the movement of the eye muscle dominated by the oculomotor nucleus, an important nuclear group of the brainstem. It has been reported that dysfunction of the neurotransmitter 5-hydroxytryptamine (5-HT) can lead to the instability of the internal environment in response to stress and plays an important role in the pathology of PTSD and that the 5-HT1A receptor (5-HT1AR) is critically involved in regulating mood and anxiety levels. In this study, the 5-HT1AR expression in the oculomotor nucleus was examined in rats with single-prolonged stress (SPS), a well established post-traumatic stress disorder animal model. Our results show that the expression of 5-HT1AR in the oculomotor nucleus neurons gradually increased 1, 4, and 7 days after exposure to SPS in comparison to the normal control group, measured by immunohistochemistry, western blotting, and reverse transcription polymerase chain reaction (RT-PCR). The expression of 5-HT1AR reached its peak 7 days after the SPS exposure and then decreased 14 days after. There is also a change in the ultrastructure in the oculomotor nucleus neuron upon SPS treatment which was observed by transmission electron microscopy. These results suggest that SPS can induce a change of the 5-HT1AR expression in the oculomotor nucleus, which may be one of the molecular mechanisms that lead to PTSD.

## Introduction

Post-traumatic stress disorder (PTSD) is an anxiety disorder that develops after exposure to a life-threatening traumatic experience. It is characterized by symptoms that patients often endure for years, including continuous re-experience of the traumatic event, avoidance of stimuli associated with the trauma, numbing of general responsiveness, and increased arousal (O'Donnell et al. 2007). The pathophysiology of PTSD has been widely studied in neuroscience (Kessler 2000), but the exact mechanism of PTSD has been elusive.

5-Hydroxytryptamine (5-HT) is an important neurotransmitter and has various functions, which include the regulation of mood, appetite, and sleep. 5-HT also has some cognitive functions, including memory and learning (Humphrey et al. 1993). Levels of the 5-HT1A receptor (5-HT1AR) are highly responsive to stress. In fact, it has been used in the treatment of mental disorders such as depression and anxiety. Several reports demonstrate that the serotonergic system plays an important role in the pathophysiology of PTSD (Lucki 1998; McAllister-Williams et al. 1998). Dysfunction of the 5-HT system can lead to the instability of the internal environment in response to stress, which may play an important role in the pathology of PTSD (HM van Praag 2004). 5-HT1AR participates in cognitive impairment and anxiety symptoms and is closely related to affective disorders (Bier and Ward 2003). The 5-HT system and the hypothalamic–pituitary–adrenal (HPA) axis, the key regulator of the stress reaction, have complex interrelationships (Porter et al. 2004). In particular, 5-HT1AR is markedly susceptible to the modulation of stress and HPA axis activation and is known to play a significant role in the pathophysiology of mood disorders (Mitsukawa et al. 2006; Cryan and Leonard 2000; Leitch et al. 2003).

The dorsal raphe nucleus is the largest nucleus of the brainstem 5-HT nuclei, containing about 50 % of the serotonergic neurons in the rat central nervous system (Wikund and Bjorklund 1980). It is estimated that the human brain has about 235,000 serotonergic neurons (Bakerk et al. 1990). The oculomotor nucleus is an important nuclear group of the brainstem. It plays a key role in the entire complex visual pathway. Dysplasia or damage of the oculomotor nucleus and the surrounding tissues could result in abnormal eye movement. Nystagmus and abnormal eye movements are being explored as diagnoses for certain diseases (Brookler 1990). It is conceivable that PTSD induces affective disorders as well as the corresponding symptoms in the oculomotor nucleus that governs the movement of the eye muscle function. A previous study showed that 5-HT1AR is expressed in the oculomotor nucleus in poultry (Huang et al. 2006). However, there have been no reports investigating the potential changes of 5-HT1AR in the oculomotor nucleus using a PTSD animal model. Herein, we aim to determine whether there are changes in 5-HT1AR in the oculomotor nucleus in the rats upon exposure to single-prolonged stress (SPS), a well-established model for PTSD (Liberzon et al. 1997; Liberzon et al. 1999; Takahashi et al. 2006; Iwamoto et al. 2007; Khan and Liberzon 2004). Using this model, our results reveal that SPS could induce a change in the 5-HT1AR expression in the oculomotor nucleus and the ultrastructure in the oculomotor nucleus neuron, suggesting a potential mechanism for the progression of PTSD.

## Materials and Methods

### Animals

Seven- to 8-week-old Wistar rats (approximately 180–200 g), provided by the Animal Experimental Center of China Medical University, were used for all experiments. The rats were housed singly in clear polycarbonate cages (46 × 24 × 20 cm = *l* × *w* × *h*) for 1 week prior to the experiments. All the rats were habituated to their cage and given standard food pellets and water. They were housed under a reversed 12/12-h light/dark cycle (lights off at 10:00 p.m) and ambient temperature (23 ± 2 °C) with humidity of 55 ± 5 %. The rats weighed approximately between 230 and 250 g on the day they were exposed to SPS. All animal protocols were carried out in accordance with the Guidance Suggestions for the Care and Use of Laboratory Animals formulated by the Ministry of Science and Technology of the People's Republic of China. All efforts were made to reduce the number of animals used and to minimize animal suffering during the experiment.

### Model Establishment and Grouping

After lab adaptation and handling, the rats were randomly assigned to one of five groups (20 in each group). One group served as a normal control, while the rest of the groups were SPS groups. The normal control rats were killed after the acclimation period, and the SPS groups' rats were exposed to the SPS procedure at the same time. The SPS model was prepared according to the previous report (Takahashi et al. 2006; Kohda et al. 2007; Liberzon et al. 1997). Each rat was restrained for 2 h by placing it inside a disposable clear plastic bag with only the tail protruding. The plastic bag was closed with tape at the base of the tail. The size of plastic bags was adjusted according to the size of the rat in order to achieve complete immobilization. A hole in the plastic bags allowed the rats to breathe freely. After immobilization, they were individually placed in a clear acrylic cylinder (240 mm in diameter and 500 mm in height), and two thirds of the cylinder from the bottom was filled with water (24 °C). The rats were forced to swim for 20 min. Following a 15-min recuperation, the rats were then exposed to ether vapor until they lost consciousness and then were left undisturbed in their home cage. Consistent with time-dependent sensitization, behavioral experiments are generally undertaken 1–14 days after the SPS procedure (Yamamoto et al. 2009). In this study, the SPS groups of animals were randomly assigned into a group 1 day after the SPS procedure (*n* = 20; SPS 1d group), a group 4 days after the SPS procedure (*n* = 20; SPS 4d group), a group 7 days after the SPS procedure (*n* = 20; SPS 7d group), and a group 14 days after the SPS procedure (*n* = 20; SPS 14d group). Each group had 20 rats, in which five rats were used for frozen sections, five for Western blotting, five for reverse transcription polymerase chain reaction (RT-PCR), and five for transmission electron microscopy (TEM).

### Measurement of Animal Body Weight

The weights of both the normal control group and SPS groups' rats were measured every other day for 2 weeks. The body weight growth curve was drawn based on the average weight in each group of rats. The body weight growth curves from the normal and SPS groups were then compared.

### Immunohistochemical Analysis of 5-HT1AR

The rats of each group were anesthetized with 10 % chloral hydrate. The hearts were exposed, and the left ventricles were perfused with 200–300 mL of 0.9 % saline via a catheter through the ascending aorta until a colorless infusion was achieved, followed by perfusion with a 300-mL fixative of 4 % paraformaldehyde (PFA; Liu 2006). The whole brains were rapidly removed and dissected on ice, followed by 6–10 h of post-fixation in 4 % PFA at 4 °C. After being immersed in 20 % sucrose solution and frozen in liquid nitrogen, coronal sections of the brain tissue were cut into slices with 12 μm in thickness and stored at −20 °C.

Sections of brain tissues were treated with 5 % bovine serum albumin (BSA) and 0.3 % Triton X-100 in phosphate-buffered saline (PBS) for 30 min at room temperature to block nonspecific staining. The sections were then incubated with goat monoclonal antibody against 5-HT1AR (diluted 1:200; Santa Cruz Biotechnology) in 2 % BSA–PBS overnight at 4 °C. After being washed three times with PBS, the sections were incubated with the rabbit anti-goat IgG (diluted 1:100; Boster) antibody for 2 h and then with the streptomycin–avidin–biotin–peroxidase complex for 1 h. The sections were washed three times with PBS. Finally, 3,30-diaminobenzidine was used as chromogen for 10 min until the brown color appeared. Slices were then dehydrated and mounted with neutral gum. As a control of assessing nonspecific staining, several sections in every experiment were incubated in PBS without a primary antibody.

Five slides were randomly selected from each group. Five visual fields of the oculomotor nucleus in each slide were randomly selected. Then, an analysis of the immunohistochemical staining positive cell rate of 5-HT1AR (immunohistochemical staining positive cell rate=immunohistochemical staining of positive cells / total cells × 100 %) was carried out, and the optical density (OD) of 5-HT1AR positive cells in each field was recorded to evaluate the average OD and analyzed using the MetaMorph/DPIO/BX41 morphology image analysis system.

### Western Blotting Used to Detect 5-HT1AR

The rats of each group were anesthetized with 10 % chloral hydrate and decapitated. The brain tissues were quickly removed and placed on ice. Oculomotor nucleus fragments were dissected from the brain tissues under a stereomicroscope in each experimental group according to the atlas of rats (Paxinos and Watson 1998). The proteins were extracted from the oculomotor nucleus samples from the normal control rats and SPS rats through homogenization, ultrasonic dispersion, and centrifugation. Samples containing 30 μg of protein were extracted. The extracted protein samples were separated by a 12 % (*w*/*v*) gradient SDS-polyacrylamide gel electrophoresis and transferred to a polyvinylidene fluoride (PVDF) membrane (Millipore, Bedford, MA, USA) using a semi-dry blotting apparatus (Bio-Rad Laboratories, Inc., Hercules, CA, USA) overnight at 4 °C. After being blocked with 5 % dried skim milk for 60 min at room temperature, the membrane was incubated with goat monoclonal antibody against 5-HT1AR (diluted 1:500; Santa Cruz Biotechnology) overnight at 4 °C. Blots were washed three times with Tris-buffered saline and Tween 20 (TBST) and then incubated with the anti-goat IgG HRP (diluted 1:5,000; Santa Cruz) for 2 h at room temperature. After the incubation, the PVDF membrane was washed three times with TBST before visualization using enhanced chemiluminescence (ECL; Amersham Pharmacia Biotech, Buckinghamshire). To confirm equal protein loading, the same blots were re-incubated with antibodies specific for β-actin (diluted 1,000; Abcam). Immunoreaction for β-actin was detected with the ECL. The OD was analyzed on a gel image analysis system. The levels of 5-HT1AR were determined by calculating the OD ratio of 5-HT1AR/β-actin.

### RT-PCR for Detection of 5-HT1AR

The rats of each group were anesthetized with 10 % chloral hydrate and decapitated. The brain tissues were quickly removed and placed on ice. Oculomotor nucleus fragments were dissected from the brain tissues under a stereomicroscope in each experimental group according to the atlas of rats (Paxinos and Watson 1998), immediately frozen in liquid nitrogen, and stored at −80 °C to be prepared for use. Total mRNA was extracted from the dissected oculomotor nucleus samples using the TRIzol kit according to the manufacturer's instructions (Takara Biotechnology). One microgram of total RNA was reverse transcribed into cDNA and then amplified using a RNA PCR kit (AM Ver. 3.0, TakaRa Bio Inc., Otsu, Japan). The specific primers were designed and synthesized by Shenggong Biotech Company (Shanghai, China) (Table 1). The reaction was started at 94 °C for 4 min; amplified for 5-HT1AR of 36 cycles for 45 s at 94 °C, 45 s at 60 °C, and 40 s at 72 °C; and ended with a 7-min extension at 72 °C. β-actin mRNA used as an internal control was co-amplified with 5-HT1AR mRNA. The PCR products were separated on a 1.2 % agarose gel by electrophoresis, and the density of each band was analyzed on a gel image analysis system (Tanon 2500R, Shanghai, China). The levels of the 5-HT1AR mRNA were normalized by β-actin.

**Table 1:** The primer sequences of 5-HT1AR and β-actin

Name	Upstream primer	Downstream primer	Product size (bp)
5-HT1A	5′-tggctttctcatctccatcc-3′	5′-ctcactgccccattagtgc-3′	357 bp
β-Actin	5-atcacccacactgtgcccatc-3′	5-acagagtacttgcgctcagga-3′	542 bp

### Assessing the Morphological Change in Oculomotor Nucleus using TEM

The rats of each group were deeply anesthetized with 10 % chloral hydrate. The hearts were exposed, and the left ventricles (Liu 2006) were perfused with 200–300 mL of 0.9 % saline via a catheter through the ascending aorta until a colorless infusion was achieved, followed by perfusion with a fixative of 2.5 % glutaraldehyde and 4 % paraformaldehyde. Then, the rats of the normal control group and the SPS groups were decapitated, respectively. The whole brains were rapidly removed and dissected on ice, and post-fixed in 2.5 % glutaraldehyde at 4 °C for 6–10 h. Then, to be chosen as a sample, the oculomotor nucleus was dissected according to the atlas of rats (Paxinos and Watson 1998) using a stereomicroscope. The dissected oculomotor nucleus samples were separated into 1-mm^3^ pieces, fixed with 2.5 % glutaraldehyde at 4 °C. The pieces were post-fixed in 1 % osmium tetroxide for 2 h at 4 °C, rinsed in 0.1 M PBS (pH 7.4) several times, dehydrated in a gradient series (20–100 %) of ethanol and then in 100 % acetone, infiltrated with Epon 812, and finally polymerized in pure Epon 812 at 65 °C for 72 h. The oculomotor nucleus was positioned in semi-thin sections. Ultra-thin sections (70 nm) were stained with uranyl acetate and lead citrate. The change in the ultrastructure of the oculomotor nucleus was examined by TEM (JEM-1200EX, Japan). A minimum of 20 sections (about 250 cells) from each group of the oculomotor nucleus was examined by TEM. Cell morphological change rates were reported as follows: cells of morphological change / total cells × 100 %.

### Statistical Analysis

All data were expressed as means±SD. Data among groups were analyzed by one-way analysis of variance using the SPSS 13.0 software. A value of *P* < 0.05 was considered to be statistically significant.

## Results

### Decreased Animal Body Weight After SPS Stimulation

**Fig. 1:**
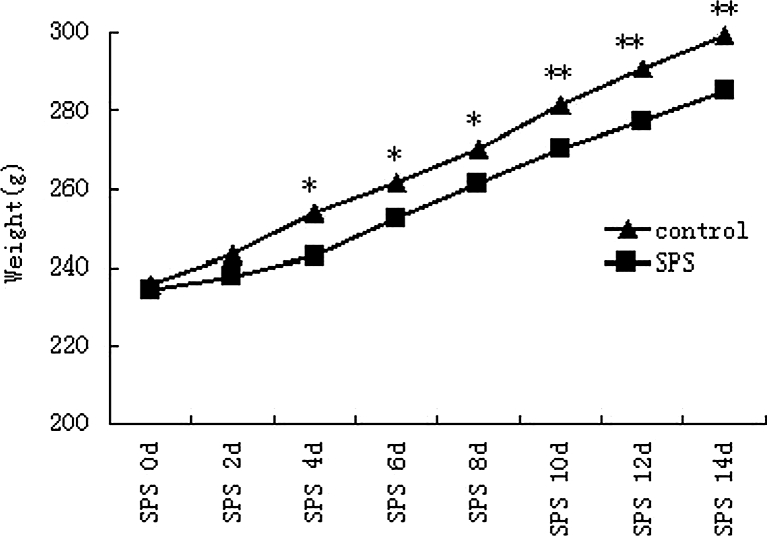
The figure is body weight growth trends in rats. Rats in the control group showed naturally increased body weight. The body weight increased slowly in the SPS groups compared with those in the normal control group. Data represent the means±SD (*n* = 5, each). *Single asterisk* denotes *P* < 0.05 vs. control group. *Double asterisks* denote *P* < 0.01 vs. control group

SPS is a well-established model for studying PTSD (Takahashi et al. 2006; Kohda et al. 2007; Liberzon et al.1997). The weights of the normal control group or SPS groups' rats were measured every other day for 2 weeks. As shown in Fig. 1, rats in the control group showed a normal increase in body weight over time. Compared with the normal control rats, rats in the model group presented weight loss after SPS stimulation (*P* < 0.05).

### Immunohistochemical Analysis Results of 5-HT1AR 

**Fig. 2:**
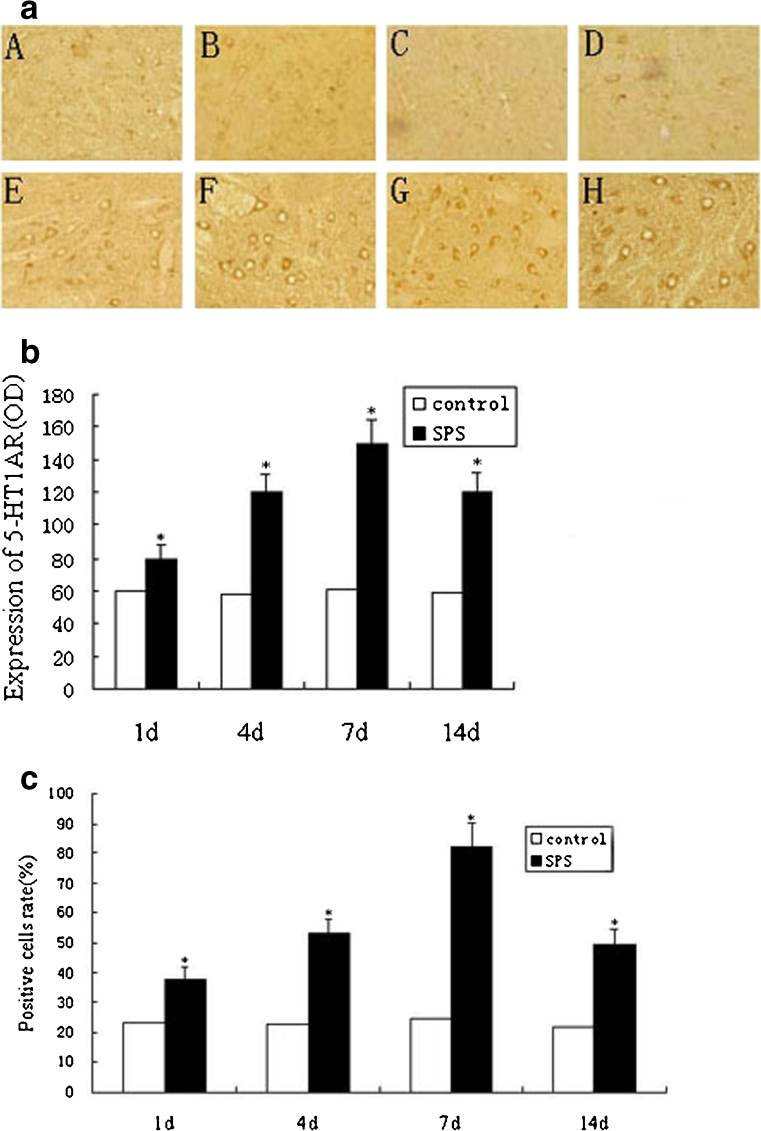
**a** Presentation of 5-HT1AR expression in the oculomotor nucleus in each group (magnification, ×200). Representative immunohistochemistry of the following groups: *A*, control 1d group; *B*, control 4d group; *C*, control 7d group; *D*, control 14d group; *E*, SPS 1d group; *F*, SPS 4d group; *G*, SPS 7d group; and *H*, SPS 14d group. **b** Mean optical densities of 5-HT1AR are shown. Data represent the means±SD (*n* = 5, each). *Asterisk* denotes *P* < 0.05 vs. control group. **c** Immunohistochemical staining positive cells rate of 5-HT1AR. Data represent the means±SD (*n* = 5, each). *Asterisk* denotes *P* < 0.05 vs. control group

After SPS stimulation, the oculomotor nucleus tissues from the treated and non-treated rats were analyzed in SPS 1d, SPS 4d, SPS 7d, and SPS 14d. The immunohistochemical staining results were shown in Fig. 2a. Positive immunohistochemical cells stained with the antibody against 5-HT1AR were brown. The immunoreactivity against 5-HT1AR was mainly observed in the cytoplasm and neurite. There were statistically significant differences in the 5-HT1AR expression among the five groups (*P* < 0.05). In the SPS groups, the brown granule in the positive immunoreactive cells had a significant increase in comparison to that in the normal control group (*P* < 0.05). The peak of the increase was in SPS 7d. Then, the immunoreactivity was significantly decreased in SPS 14d compared to that in SPS 7d (*P* < 0.05). Mean optical densities of 5-HT1AR are shown in Fig. 2b. The immunohistochemical staining positive cell rate is shown in Fig. 2c.

### Western Blotting of 5-HT1AR

**Fig. 3:**
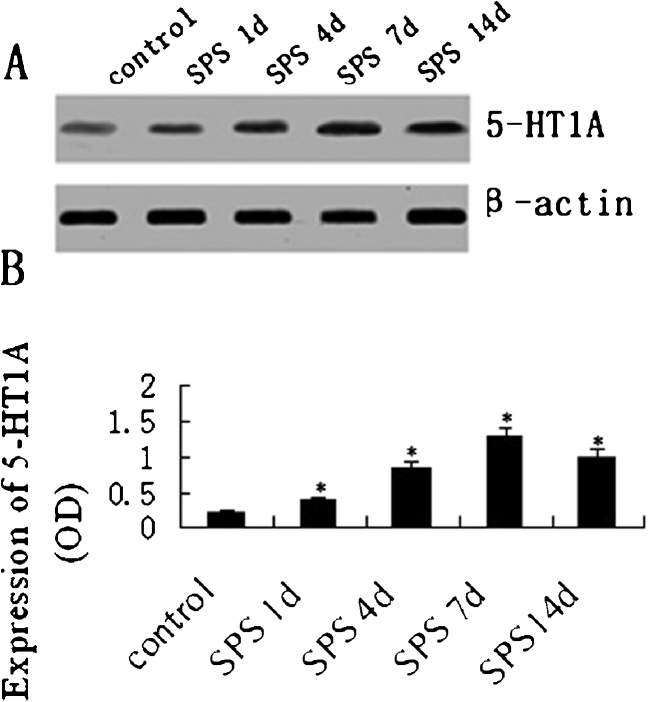
5-HT1AR expression in the oculomotor nucleus by Western blotting (*A*). Bands show 5-HT1AR protein levels. From *left* to *right*, the lanes are the following groups: control group, SPS 1d group, SPS 4d group, SPS 7d group, and SPS 14d group. β-Actin served as a loading control. Relative quantitative levels of 5-HT1AR (*B*). Data represent the means±SD (*n* = 5, each). *Asterisk* denotes *P* < 0.05 vs. control group

Western blot was used to detect the protein expressions of 5-HT1AR (Fig. 3). The 5-HT1AR and β-actin proteins were detected at 56 and 42 kDa, respectively, and the mean values of the band densities of the control group were set as 100 %. Data were expressed with normalized OD. The 5-HT1AR protein expression in the SPS groups showed a marked increase compared to that of the control group (*P* < 0.05). The peak increase occurred in the SPS 7d group. The density of the 5-HT1AR protein expression had a significant decrease in the SPS 14d group. The time course of the Western blot results was consistent with the findings obtained by immunohistochemical analysis.

### RT-PCR Results of 5-HT1AR mRNA

**Fig. 4:**
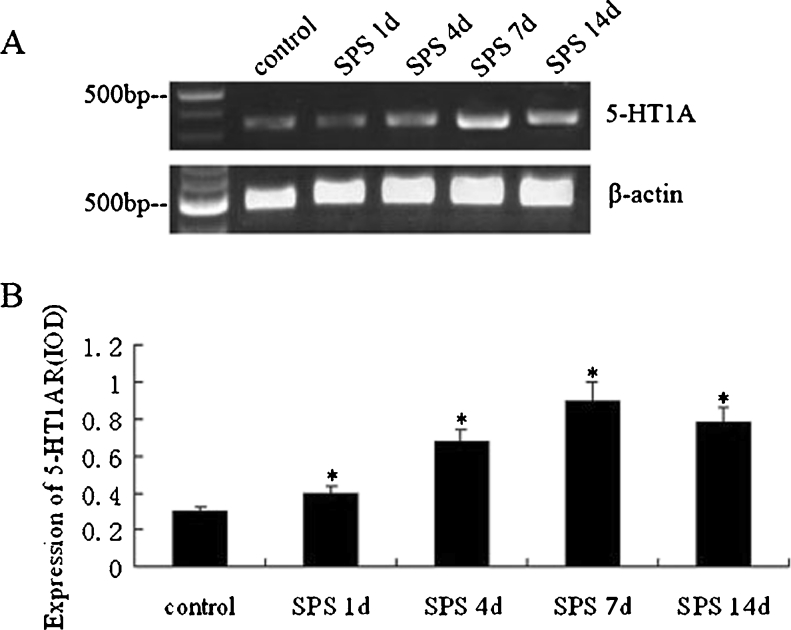
RT-PCR was used to detect changes in the mRNA expression of 5-HT1AR at the different time points after SPS stimulation (*A*). From *left* to *right*, the PCR bands are the following groups: *lane 0*, marker; *lane 1*, control group; *lane 2*, SPS 1d group; *lane 3*, SPS 4d group; *lane 4*, SPS 7d group; and *lane 5*, SPS 14d group. β-Actin served as a loading control. Relative amounts of 5-HT1AR mRNA (*B*). Data represent the means±SD (*n* = 5, each). *Asterisk* denotes *P* < 0.05 vs. control group

To futher confirm the increase in 5-HT1AR expression by SPS, we performed RT-PCR analysis (Fig. 4). The levels of 5-HT1AR mRNA were normalized with β-actin mRNA. The levels of 5-HT1AR mRNA increased significantly in the SPS groups compared to those in the normal control group (*P* < 0.05) and peaked in SPS 7d. The change at the protein levels observed above and the levels of 5-HT1AR mRNA which declined at day 14 in comparison with SPS 7d (*P* < 0.05) are consistent with the results of immunohistochemistry and Western blot.

### Morphological Change of the Oculomotor Nucleus by TEM

**Fig. 5:**
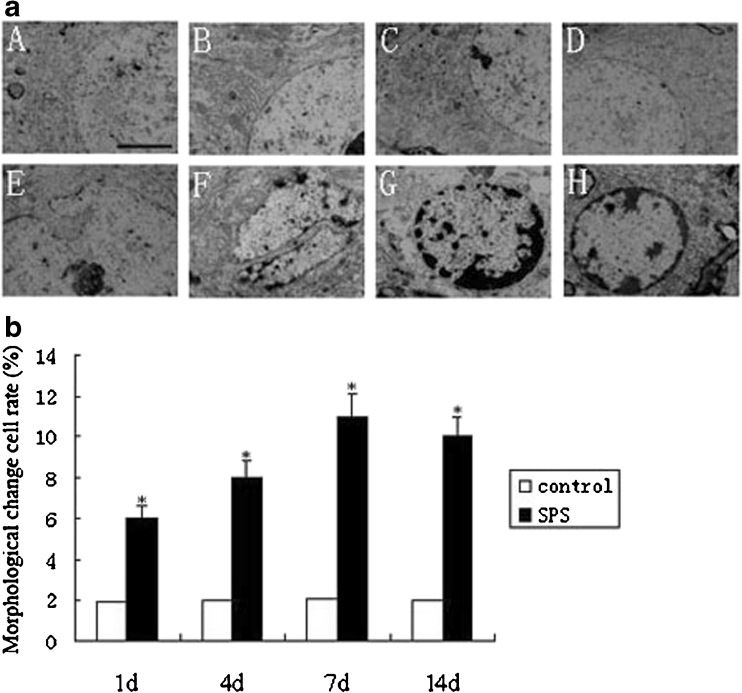
**a** The oculomotor nucleus neurons of rats in each group under a transmission electron microscope. *A*, control 1d group; *B*, control 4d group; *C*, control 7d group; *D*, control 14d group; *E*, SPS 1d group; *F*, SPS 4d group; *G*, SPS 7d group; and *H*, SPS 14d group. *Scale bars* = 500 nm. **b** Cell morphological change rate was calculated by TEM at different time points. Most cells have morphological and structural changes in SPS 7d. Data represent the means±SD (*n* = 5, each). *Asterisk* denotes *P* < 0.05 vs. control group

As shown in Fig. 5a, the oculomotor nucleus neuron exhibited a normal structure in the control rats (Fig. 5a, A–D). However, some neurons in the SPS group rats showed an irregular nucleus shape and partially reduced cristae of the mitochondria (Fig. 5a, E–H). The results also showed chromatin condensation, appearance of chromatin crescents, and a partially dissolved cytoplasm (Fig. 5a, G). Furthermore, these changes were observed mostly in SPS 7d (*P* < 0.05), which are consistent with the results of immunohistochemistry, Western blot, and RT-PCR. The rates of the cell morphological change were reported in Fig. 5b (*P* < 0.05).

## Discussion

PTSD is classified as a type of anxiety disorder (American Psychiatric Association 1994). In recent years, the incidence rate of PTSD, which has been seriously endangering the people's physical and mental health, has increased. The prevalence of PTSD in the general population is approximately 8 % in the USA (Vieweg et al. 2006; Yehuda and LeDoux 2007). The nature of the trauma is an important factor to the risk for developing this disorder. A new study about the psychological reactions of survivors of the 512 Wenchuan earthquake in China suggests that PTSD symptoms affected 84.8 % of the survivors 1–2 months after the earthquake (Zhang and Ho 2011). An enormous disaster makes mental stress so strong and long lasting that it leads to the damage of the memory loop, excitement of the central nervous system, and inhibition of the expression of neurotransmitters, which futher lead to the formation of PTSD.

The biological mechanisms underlying PTSD are chiefly involved in three aspects: a reduced function of the corticosteroid receptor, noradrenergic and pressure element-excited system, as well as the 5-HT system's shortcomings (HM van Praag 2004). As the serotonergic system plays an important and generalized role in the regulation of sleep–wake states and behavioral arousal, selective serotonin reuptake inhibitors are widely used for the treatment of PTSD. 5-HT is involved in the occurrence of PTSD by interacting with the 5-HT transporter, 5-HT receptor (including 5-HT1A, 5-HT1B, 5-HT2A, and 5-HT2C receptors), and the neurotransmitters dopamine and norepinephrine. The 5-HT positive cells are widely distributed in the mammalian tissues, especially at high levels in the cerebral cortex and synapses, and are inhibitory neurotransmitters (Gillman 2009).

Studies have shown that neurotransmitter imbalance, in particular, the abnormal level of 5-HT abnormalities, is closely associated with PTSD (Zhang et al. 2010). 5-HT is involved in a variety of physiological and pathological processes, mediated by over 14 types of receptors. Among these receptors, the 5-HT1AR receptor is the most abundant in mammalian brain tissues (Aznar et al. 2003). 5-HT1AR can couple with Gi to inhibit adenylate cyclase and activate cytomembrane K^+^ channels, or couple with Ca to shut down the Ca^2+^ channel. 5-HT1AR is not only the presynaptic receptor, but also the post-synaptic receptor, and both the presynaptic and post-synaptic receptors work in conjunction (Albert and Lemonde 2004). 5-HT1AR is an important regulator in 5-HT neurotransmission (Feng et al. 2001; Muller et al. 2007). 5-HT1AR is also involved in the regulation of the HPA axis during stress response (Carrasco and Van de Kar 2003). Dysfunction of the HPA axis function is the key for neuroendocrine abnormalities during PTSD (Wang et al. 2009). Rats exposed to SPS exhibited enhanced inhibition of the HPA system and alteration of the glucocorticoid/mineralocorticoid receptor. A behavioral study further showed that PTSD can increase the 5-HT1A receptor density (Zhang et al. 2010). 5-HT1AR has been closely linked to human anxiety, depression, schizophrenia, pain, cognitive ability, eating behavior, sexual activity, Alzheimer's disease, and the sleep–wake cycle (Muller et al. 2007; Aghajanian and Sanders-Bush 2002; Nichols and Sanders-Bush 2001).

The oculomotor nucleus is located in the ventral part of the aqueduct of the midbrain with an irregular quadrilateral or oval shape and is separated from the aqueduct interval by white matter fibers and the gray matter. The oculomotor nucleus is an important nucleus in the brainstem and plays a key role in the entire visual pathway. The nerve fibers come from the oculomotor nucleus which controls the superior rectus, inferior oblique, medial rectus, inferior rectus, and palpebralis of the eye. In the meantime, the oculomotor nucleus also receives bilateral cortical fibers and vestibular fibers of the compound nucleus and thus forms a reflex arc for the coordinated movement of the head and eye.

Our previous study reported that there is an increase of 5-HT1AR levels in the dorsal raphe nucleus in the rats subjected to SPS (Luo et al. 2011). Thus, we postulate that there may be changes of 5-HT1AR levels in the oculomotor nucleus after SPS stimulation, which may contribute to the dull eye symptom in PTSD patients. In this study, we found that SPS stimulation indeed increases the expression of the 5-HT1A receptor in rat oculomotor nucleus neurons. 5-HT1A receptors are located in the neuron cell body and neurite of the oculomotor nucleus and are the receptors of the presynaptic membrane. Based on our finding, we postulate that the increase in the expression of 5-HT1AR upon SPS stimulation results in an enhanced negative feedback regulation of the 5-HT nervous system, which inhibits the electrical activity of 5-HT neurons in the oculomotor nucleus, and further results in a decrease in biosynthesis, transport, and release of 5-HT. It has been reported that Ca^2+^ overload and disorder of the Ca signal regulation in the nerve cells in PTSD patients increased the cytotoxicity of the brain neurons (Xiao et al. 2009). Our finding that there is an increased expression of the 5-HT1A receptor in neurons of the oculomotor nucleus upon SPS stimulation suggests that the 5-HT1A receptors in the post-synaptic neurons of the oculomotor nuclear membrane stimulated the adenylate cyclase system, reduced the activity of adenylate cyclase, interfered with the signal transduction pathway, inhibited neuronal hyperpolarization, and resulted in a decreased cytoplasmic Ca^2+^ concentration. This leads to the opening of the Ca^2+^ channel, resulting in an influx of extracellular Ca^2+^ into the cell. Overload of intracellular Ca^2+^ can activate the myosin ATPase of the eye muscles, which promotes ATP hydrolysis and supplies energy, leads to the sliding of the filaments to the middle of the sarcomere, and causes eye muscle fiber contraction. In addition, an increase in 5-HT1A may also explain the increased alertness in PTSD patients, which has been reported to be associated with increased norepinephrine levels (Zhang et al. 2010). The 5-HT1A receptor of the post-synaptic membrane may affect the release of norepinephrine. The SPS-induced increase of 5-HT1A receptors in neurons of the oculomotor nucleus may reduce the 5-HT neurotransmitter released from the oculomotor nucleus to control the eye muscle nerve fibers but may increase norepinephrine release, which leads to increased alertness, anxiety, and startle reactions accompanied by the symptoms of abnormal eye movement in PTSD patients.

Because the nerve fibers of the nuclear oculomotor that control the medial rectus and inferior rectus come from both sides of the nuclear oculomotor, the extraocular and the intraocular muscles are often not being stressed at the same moment, which is often presented as an incomplete injury. Eye movement is accomplished by contraction of the extraocular muscles. PTSD patients often have symptoms of extraocular oblique muscle disorders, inward or vertical eye movement disorders, and ptosis. Our findings in this study suggest that SPS stimulation increases the 5-HT1A receptor levels of the oculomotor nucleus, which may lead to dull eyes in PTSD patients. The observed ultrastructural changes of the nucleus, cytoplasmic mitochondria, and rough endoplasmic reticulum which appeared in the oculomotor neurons of the rats after SPS exposure may directly affect the energy metabolism and protein synthesis in the neurons, leading to the secondary damage of the neurons.

In conclusion, our results showed that SPS induces an increase of the 5-HT1AR expression and morphological change of the neurons in the oculomotor nucleus, which may be one of the important pathophysiological bases for dull eyes and the anxious, frightened, and other abnormal emotional behavior in PTSD rats. Overexpression of 5-HT1A receptors may play an important role in the development of PTSD.
